# Understanding the value of dietary indices prior to research application

**DOI:** 10.1186/s12916-021-01938-1

**Published:** 2021-03-17

**Authors:** Mary Foong-Fong Chong

**Affiliations:** 1 Saw Swee Hock School of Public Health, National University of Singapore and National University Health System, Singapore, Singapore; 2 Singapore Institute for Clinical Sciences, Agency for Science, Technology and Research, Singapore, Singapore

**Keywords:** Dietary quality indices, Diet inflammatory index, Meta-analyses of individual participant data

## Background

Diets are complex to examine as they are multi-dimensional and often accompanied by internal correlations. The use of dietary indices to quantify dietary quality and/or variance via a single, simplified measure has gained traction in the last two decades. Several diet indices have been developed to evaluate the role of diet in relation to chronic disease mortality, with most originally based on data from US and Europe populations. Following which, a number of studies have emerged to investigate the compatibility and applicability of various indices for different health outcomes and for different population groups. Current evidence suggests that certain indices maybe more suitable for examining certain health outcomes or populations, as different indices explain different variance in food intakes and/or that certain dietary components captured may not be as relevant to the investigated health outcomes or cultural context [1, 2].

## Assessing maternal diet quality and its impact on child obesity

The study by Chen L-W et al. brings to mind this pertinent point, but also brings to the table a refreshing perspective of the value of using different dietary indices to understand the various mechanisms diets may act through, in relation to health [3]. By assessing maternal diets using a diet quality index (the DASH score) and the Diet Inflammatory Index (DII) in relation to child adiposity, Chen L-W et al. elegantly demonstrated that maternal diet quality was more strongly related to the global measure of adiposity (BMI cutoffs) when compared to the diet inflammation score, whereas the latter was more closely associated with child’s body composition (fat-free mass and fat mass) [3]. Through additional mutual adjustment and mediation analyses, they were able to conclude that while the inflammatory components of diet may in part explain the relationship between diet and childhood adiposity, overall diet quality and/or other components of diet also have their roles in influencing childhood adiposity, but likely through other mechanisms (e.g., diet-induced modifications of epigenes). Indeed, this study provides an important reminder to understand the value and predictive ability of dietary indices prior to selecting one for investigating diet in relation to specific health outcomes of interest [3]. The value of a dietary index is only as good as its components and the contribution of the components to the calculation of the total score. Dietary indices can serve different purposes; thus, selecting an appropriate index that is meaningful to answer one’s scientific hypothesis is critical.

The dietary index of particular interest here is the DII, which has been applied to various clinical conditions, but only more recently been extended to the area of maternal and child health. The DII was developed and validated to characterize and quantify the cumulative inflammatory potential of individual diet. One of its key advantages is that it measures the standardized contribution of a wide variety of foods and nutrients based on inflammatory biomarkers objectively measured in cell, animal, or human studies. This allows it to be universally applied across many cultural contexts and derived from different dietary assessment tools, thus allowing comparison of studies globally [4].

While the relationship of maternal or early life pro-inflammatory diets (measured using the DII) and child adiposity has previously been examined in two other cohort studies with approximately 1000 mother-child pairs each [5, 6], a real strength of the current study is its significantly large sample size of *n* = 16,295 mother-child pairs—derived from using individual participant data pooled from seven European cohorts in the ALPHABET consortium. As opposed to traditional meta-analysis, the commendable effort in harmonizing data across multiple studies would have reduced substantial heterogeneity across studies due to incomplete or selective reporting and consequently facilitated more accurate interpretation of results. Adequately powered sensitivity analyses were also conducted, thus increasing the robustness of the findings.

## Conclusion

Meta-analyses are an integral part of evidence-based health research and whilst it is recognized that individual participant data meta-analysis offers numerous clinical and statistical advantages, one should be cognizant of its multiple challenges of being resource-intensive, the need for advanced statistical expertise and intensive co-ordination across groups/studies, amongst other challenges, before embarking on one [7]. Replication and comparative studies across cohorts may be a good compromise, as comparing cohorts from different ethnicities, physical environment, and social and cultural settings invariably provide valuable insights. Future research studies should consider the use of more than one dietary index for different purposes, so that together they add different dimensions to answering the scientific question.

## Data Availability

N.A.
